# Effectiveness of a mindfulness-based program with virtual reality to increase safe behaviors in workers of a mining company

**DOI:** 10.3389/fpsyg.2025.1429334

**Published:** 2025-03-14

**Authors:** Raúl Guzmán, Yuri Félix Chávez-Luque, Nisida Guzmán, Guillermo Sebastián Medina, Celin Daniel Valdiviezo, Alejandro Santa-Cruz

**Affiliations:** Catholic University of Santa María, Arequipa, Peru

**Keywords:** mindfulness, virtual reality, work accidents, BBS, risk behaviors

## Abstract

**Objectives:**

The development of safe behaviors is a transcendental issue, especially in organizations where jobs considered high risk, such as mining, are carried out. This study proposes a program aimed at increasing safe behavior through a mindfulness program supported by virtual reality techniques. The specific objective was to determine the effect of this program on safe behaviors, comparing with those produced with the BBS (Safety Based On Behavior) program in a sample of workers who performed high-risk activities in a mining company in southern Peru.

**Method:**

Based on the determination of high-risk activities for the study, the study groups were randomly selected, forming two equivalent groups in terms of sex, age, education, and marital status. The study units were randomly assigned to one of the following 2 groups: 22 to the control group (BBS) and 22 to the experimental group (mindfulness) to whom record sheets of safe behaviors related to standard risk behaviors were applied for each activity on a weekly basis, according to the BBS system where the observers did not know the composition of the groups under study.

**Results:**

Data analysis showed evidence of a significant improvement in the index of safe behavior thanks to the proposed mindfulness program, compared to the BBS program alone.

**Conclusion:**

Evidence was found that the mindfulness-based program significantly reduces the number of risk behaviors likely to cause workplace accidents, maintaining that mindfulness is a very useful tool to reduce the number of incidents and/or accidents in workers. An organization, in this case, a mining company.

## Introduction

Today, all organizations consider the health and safety of their workers to be a priority. To achieve this, it is not only a matter of transmitting knowledge or motivations about safety, but also of generating changes in safe behavior in such a way that its long-term sustainability is ensured. This implies the need to produce evident changes in behavior so that safety is part of a culture (Pereira et al., 2023), supported by specific changes in behavior and attitudes toward the prevention of occupational risks, which allows the effective prevention of occupational accidents (Soto and Mogollón, 2005).

In recent years, mindfulness has found important support in neuroscience through multiple studies on changes in brain structure and processes (Tang et al., 2015), as well as its practice has been demonstrating a valuable contribution in a variety of areas of human activity such as health, education, sports and more recently in the prevention of occupational risks and accidents, particularly in the performance of high-risk tasks such as those carried out in the mining and industrial fields. In this environment, mindfulness applied through programs offers important benefits for the health and well-being of workers, as well as for occupational safety, as it demonstrates its usefulness in the prevention of physical and psychosocial problems such as stress, depression, eating disorders, and anxiety (Góngora and Castro, 2015). Beyond this, mindfulness awareness and education help empower supervisors and managers, providing them with training that can support and encourage practice in their teams; these programs also highlight the importance of mindful leadership and effective stress management in demanding work environments, while fostering an organizational culture that appreciates the importance of employees’ mental and emotional well-being (Skovbjerg et al., 2023).

In particular, we find the valuable contribution of mindfulness in the prevention of occupational risks and accidents in organizations, allowing us to reach an alert situation about some details about the mental processes involved in the regulation of behavior at work (Vogus and Sutcliffe, 2012). Specifically, mindfulness acts to improve metacognitive capacity and executive attention, improving productivity and the prevention of occupational risks (Vogus and Sutcliffe, 2012). As evidence, it was found that workers with better development of mindfulness skills showed fewer safety problems; it was also identified that the lack of mindfulness is associated with errors in safety regulations (Liang et al., 2022). In summary, mindfulness programs reduce risky behaviors (Zhang and Wu, 2014).

It is also important to note that mindfulness promotes greater awareness of the stimuli present in the environment, which can help improve concentration on crucial tasks, reducing the likelihood of distractions that can lead to accidents or mistakes (Zeidan et al., 2010). Therefore, a regular mindfulness practice is associated with reducing stress and improving cognitive functions, which can contribute to a better emotional state and therefore improve decision-making in critical situations, such as those experienced in risky work environments (Goyal et al., 2014).

As a safety training tool, mindfulness is being used in some work contexts, including high-risk sectors such as mining, as a training tool. These interventions aim to teach workers how to develop greater awareness, attention, and emotional control, skills that can have a direct impact on reducing accidents and improving safety (Vickers and Vogt, 2014). Whereas, by promoting a culture of safety, implementing mindfulness practices within a mining organization can foster a more proactive safety culture. Workers and leaders can be more aware of their own actions and the effects they have on overall safety (Houtman and Kompier, 2016). This can improve communication, collaboration, and commitment to safety regulations, creating a safer work environment (Houtman and Kompier, 2016).

Regarding the relationship between mindfulness and work stress, people with a high level of mindfulness focus their attention on the present moment, instead of having their mind focused on circumstances beyond their control, mindfulness allows us to differentiate between the characteristics of the environment and the reactions that one has to them by dissociating their reactions from the environment; this implies that they separate the recognition of stressors in the environment from their automatic reactions to those stressors (Vickers and Vogt, 2014). In this way, workers turn to more efficient coping strategies to reduce stress (Goilean et al., 2020). Likewise, awareness of the present moment allows for better adaptive responses to stress, regardless of the individual’s affective state and the severity of the threat experienced (Donald et al., 2016). Some mindfulness-based programs have already been shown to be effective in reducing stress, such as MBSR, recommended in high-risk jobs with a significant level of stress (Molek and Żołnierczyk, 2018), on the other hand, mindfulness is a predictor of safe work behavior (Valley and Stallones, 2017). Another reported benefit is that mindfulness decreases emotional exhaustion and increases job satisfaction (Hülsheger et al., 2013).

These examples demonstrate that mindfulness can be considered a valuable tool in the health and well-being of workers in mining organizations, as it strengthens *engagement* by allowing workers to see work activities in a more innovative and interesting way. An employee with a high level of mindfulness development will easily identify when they are distracted and will be motivated to refocus and continue to be “engaged” in the workplace. The present moment, allowing him to return to the task; it can also help reduce distress, reporting an increase in the level of satisfaction, in addition to optimizing the worker’s breathing rate and heart rate. These results show the important role of mindfulness in psychological well-being and the reduction of workplace accidents, while helping us to understand the increase in the application of mindfulness programs in organizations (Daigle et al., 2018).

Mindfulness is “an unbiased awareness that is generated moment by moment through open attention that is non-reactive and free of prejudice in the present moment” (Kabat-Zinn, 2003). As such, the mindfulness program applied in the mining company has five components such as: “attention to the moment,” which implies the fact that the individual must focus on the present moment, instead of being focused on the past or the future, since attention must be focused on the internal processes of each individual, as well as in the actions of their daily lives. This attention can change depending on the moment and its importance. Another component is “openness to experience,” which is the ability to observe an event without interjecting beliefs; that is, observing situations as if it were the first time, a quality that is called “beginner’s mind,” where the individual must be open to the curiosity of wanting to know more. A third component is “acceptance,” in which we are told that in order for the individual to experience full awareness, it is necessary to opt for an attitude of acceptance without judging the thoughts, feelings, and events of everyday life. The fourth component is “letting go” which refers to the fact that the individual should not allow himself to be held back by any thought, feeling, sensation or desire, nor to be turned off, much less identify with them; Because it is mistakenly believed that the more you achieve in material things, the happier you are. Therefore, it is necessary for the person to let go or let go of something that he clings to so much. The fifth component is “intention,” which states that every action must have a purpose, it may not be immediate, but each individual must move based on a personal purpose (Pérez and Botella, 2006; Vásquez-Dextre, 2016). Likewise, some techniques used were “breathing,” which becomes a fundamental element of meditation, allowing you to be aware of your own thoughts; “Guided Visualization,” which is linked to mental representation to be able to visualize oneself in an environment or also the visualization of an object, for this it is necessary to use the five senses. Finally, “guided body exploration,” which means focusing attention on one’s own body, paying attention to its bodily sensations, feeling, identifying and understanding each stimulus in each part of the body with the aim of participating in the body state while inducing relaxation (Gómez et al., 2019; Luis-Pascual, 2016).

Recently, the benefits of the relationship between mindfulness and the use of virtual reality have been documented, as a facilitator of the process of achieving mindfulness where the feasibility and acceptability of virtual reality to facilitate the practice of mindfulness is evaluated (Navarro-Haro et al., 2017). In the workplace, virtual reality has been used since the beginning of the century, in aspects related to organizational development, such as improving training and learning (Riva et al., 2007), increasing productivity and work efficiency (Pan et al., 2019), reducing the costs and risks associated with training and work (Chen and Wang, 2016), or stimulating creativity and innovation (Qian and Clark, 2016). In this study, we decided to incorporate virtual reality as a support for mindfulness in the improvement of behaviors related to safety and health at work.

## Method

### Participants

The participants came from a mining company with approximately 126 workers. With the authorization of the company and under its supervision, 2 groups of 22 workers were formed that were homogeneous, the criteria to form the blocks were sex, profession, educational level, marital status. The final sample consisted of 44 workers divided into 2 groups; the control group (CBS) was composed of 22 workers with a mean age of 37 years (SD = 8.994), 90.9% (20) men and 9.1% (2) women, 45.5% (10) employees and 54.5% (12) workers. The workers belonged to different areas such as: administration and human resources, kitchen, maintenance, mine, planning, plant, heritage protection and security. The experimental group (mindfulness) was also composed of 22 workers, with a mean age of 39 years (SD = 7,462), 95.5% (21) men and 4.5% (1) women, of whom 27.7% (5) were employees and 77.3% (17) workers. All participants completed the research, there was no dropout or dropout in either group. Nor was any qualitative assessment applied to participants’ perceptions of mindfulness. The registry was a checklist that recorded the behaviors of the participants, a baseline and a post-evaluation were carried out up to 5 weeks after the end of the application of the program to complement the evaluation over time and see if the effects of the Mindfulness Intervention Program were still present. Some of these limitations were due to directives from the mining company, nor have replications of the program been made in the company since the application was in charge of the team of researchers.

### Procedure

A mindfulness-based program was developed to compare it with a control group, which applied a program based on BBS (behavior-based safety), which is usually used in mining companies as an alternative to improve and increase safe behaviors. It is important to mention that the group that was given mindfulness continued to use the BBS within what was normally established by the organization: The total duration of the application of the program was 9 weeks. Likewise, the mindfulness-based program was complemented with virtual reality, in addition to meditation tasks that were left as activities for each participant to do at home. A 4-week baseline was developed to initiate program implementation and then the 9-week program was initiated; to finally follow up for 5 weeks after the application of the mindfulness program.

The mindfulness-based program employed techniques such as mindful breathing, guided imagery, and guided body scanning; use the resources and materials prepared for each session, but it also used virtual reality equipment and programs (sessions 3, 4, and 6). It had 9 sessions of 30 min in duration in each session. The sessions were divided into: motivation, exploration and problematization (5 min), knowledge construction (10 min), transfer (10 min), evaluation (3 min) and closure (2 min). In session 1, an introduction to mindfulness was made, concepts about mindfulness were explained, generating interest in the topics. In session 2, guided body visualization and exploration were worked on, seeking to learn about the principles and procedures, as well as their application. In session 3, “Relaxation and observation of stressors” was worked on with virtual reality, seeking to raise awareness about the effects of stress, sharing knowledge on how to control it and with exercises that were practiced. In session 4, virtual reality continued, “body exploration and active pause” was worked on, the objective was to understand the fundamentals of active pauses and their relationship with mindfulness, in a practical way. In session 5, we worked on the “Application of mindfulness to the awareness of thought processes and walking meditation” seeking to understand the benefits of walking meditation, relating it to the other techniques learned in a practical way (VR was not done in this session). Session 6 was the third and final session with virtual reality, working on the “observation of stressors and their physiological correlates, accompanied by a relaxation response,” with the aim of achieving an understanding of the advantages of applying relaxation strategies in a practical way. In session 7, we worked on “Seated meditation and guided body exploration.” In session 8, “Concentration and fluency in work tasks” was worked on with the aim of understanding and applying concentration and fluency at work. In session 9, we worked on “The integration of mindfulness in daily life/closure,” so that they have a global knowledge of everything taught and that this is integrated into the work.

With respect to virtual reality, there is currently an increase in the use of equipment for purposes based on virtual reality (VR) applications such as the application of mindfulness. Virtual reality can be influenced by technological differences such as resolution, refresh rate, and content; this is relevant for future research (Saredakis et al., 2020). In our studio, in order to simulate an ideal space, we used the “Nature Treks” program, where meditation can be recreated in a space in contact with nature, which allowed us to explore from snow-capped mountains to the stars. To standardize the virtual reality sessions, the “Green Meadows” map was used, which provided interaction with living nature, a giant field of vegetation and views of the mountains, being a perfect setting for meditation, the use of this program is only a complement or an additional to meditation, it serves in effect so that the person can visualize the environment in real time instead of imagining it. Likewise, the virtual reality equipment called HTC VIVE COSMOS ELITE from the “VIVE” brand, a company in charge of developing hardware, software and everything related to the metaverse, was used. The acquisition of the equipment contains various accessories (virtual reality headsets, virtual reality controllers, base station sensors, headset and laptop adapter connection, controller and sensor chargers, user manuals) that allow access to virtual reality through a high-end “gamer” laptop that can support the graphics that are displayed once the entire system is installed and the program is run on the STEAM platform or VIVE PORT. The virtual reality administration per session lasted 30 min per group and was administered in a total of two sessions of the program.

The study was approved by the Ethics Committee of the Universidad Católica Santa María and by the management of the mining company, after selecting the sample, the workers who were chosen to be part of the groups were invited, they were asked for informed consent as evidence that they agreed to participate in any of the programs to which they were selected, they were evaluated for 4 weeks; this being the baseline. The application of the program lasted 9 weeks and after this it was evaluated 5 weeks after the end of the program. Participation was voluntary and data was collected through weekly observation cards that are applied by several people and that record safe behaviors as part of the BBS’s behavior-based safety system, it is important to mention that the application of the program was developed and applied from the human resources office, since it had the collaboration of the Human Resources team.

The so-called safe behavior observation letter was used as recording measures, for which the mining company has developed an *ad hoc* observation sheet where the safe behaviors of all personnel are recorded. The recording of the behaviors is done blindly, that is, those who record the mine behaviors do not know who belongs to the control group and who belongs to the experimental group; the total score is the sum of the safe behaviors recorded, This observation sheet records safe and risky behaviors: safe behaviors are scored as 1 and risky behaviors are scored as 0. The observation sheet consists of 39 items and are as follows:

1. Manual lifting of loads: lifting, reaching, dragging or transporting.
2. Ergonomics: body mechanics, repetitive movements, posture suitable for the task, etc.
3. Entrapment points, sharp edges, pinch points, crushing and hot surfaces.
4. Face and eye protection: safety glasses, face shield, sunscreen, etc.
5. Head protection: helmet wear, chinstrap.
6. Hand protection: use of gloves in good condition and specific.
7. Protection against falls from height: use of harnesses, belts, ropes, anchor lines, retractable drums, etc.
8. Hearing protection: use of earplugs, cup protector.
9. Respiratory protection: use of masks, bidirectional respiratory screening, etc.
10. Body protection: use of shoulder pads, knee pads, leather clothing, tibex, etc.
11. Confined space protection: self-rescuer, flashlight.
12. Protection of the feet: use of shoes, boots, metatarsals, etc.
13. Line of fire: stay away from suspended loads, projected particles, moving equipment, etc.
14. There is a viewpoint and/or control pointer, its indications are respected, they have a communication radio.
15. Respects distance and signaling with moving vehicle/moves away from equipment blind spots.
16. Exceeding the permitted speed limits.
17. Delimit and signal (cones, blocks, etc.) when leaving the unit.
18. Wear the seat belt, if you are transporting personnel, you can see that they are wearing the same seat belt.
19. Turn off and lock your computer when you turn it down.
20. Selects and inspects tools and/or equipment, uses them well and maintains them.
21. Selects and inspects ladders, uses them well and maintains them, uses three points of support.
22. It has a communication radio if its function warrants it.
23. Scaffolding: inspection, condition, selection, use and storage.
24. Activity conditions: illuminated area, suitable weather conditions.
25. Pedestrian accesses; defined, free of obstacles, clear work area, etc.
26. Barricades/barriers/signs/traction tapes/cones: complete, defined, maintained and with an updated file.
27. Order and cleanliness; debris, waste collection and segregation, has drains, etc.
28. Hazardous materials: transport, storage, labeling, labeling, trays and anti-spill kits, etc.
29. IPERC, JSA: signed, approved, disseminated and visible in the work area.
30. Competent personnel: accredited to perform high-risk work. You have the proper permissions.
31. Additional permits: checklist, training card, MSDS Sheets, etc.
32. Maintain social distance (1.5 m) from person to person.
33. Wash your hands at the entrance, exit: dining rooms, bathrooms.
34. Wear your protective mask at all times.
35. When sneezing and/or coughing, cover your face with your forearm.
36. Has attachment to the activities they perform.
37. Is focused on the activity they are doing, uses cell phones at inappropriate times and places.
38. Teamwork: coordination and communication.
39. He goes about his business in a hurry.

These cards were applied to all workers on a mandatory basis and were carried out by several people in charge of recording these behaviors who had previously received training in behavior recording according to the Behavior-Based Safety (BBS) work model.

### Data analysis

In order to detect the differences between the two groups, mean difference analyses (Student’s *t*) were performed. For this analysis, we first checked Student’s *t*-assumptions, such as the Shapiro–Wilk normality test *p* ≥ 0.05 and the homogeneity of variances with the Levene test *p* > 0.05 (Tabachnick and Fidell, 2001). The means of the two groups in the pre-test and in the post-test were compared. It is necessary to mention that the mindfulness group, in addition to receiving mindfulness training, does not stop receiving BBS’s, since they are part of the mining company’s safety policies. To assess the magnitude of the program’s outcomes, effect sizes were calculated using Cohen’s *d*; values between 0.20 and 0.40 were considered small effect sizes, values between 0.5 and 0.7 moderate effect sizes, and values greater than 0.8 represented large effect sizes (Cohen, 1992; Durlak, 2009).

## Results

Student’s *t* was used to analyze the differences between the two groups (control and mindfulness) in the evaluation of safe behaviors. The results of Table 1 show that there were no statistically significant differences in the control group, which was found in the experimental group to which the mindfulness-based program was applied, this shows that mindfulness is effective in the development of safe behaviors.

**Table 1:** Analysis of the differences between pre- and post-test in safe behaviors in the BBS and mindfulness groups.

Subscale/group	*N*	Pre test		Post test				
		*M*	SD	*M*	SD	*t*	*p*	*d* Cohen
BBS group	22	88	42.98	100	15.68	1.114	0.265	0.244
Mindfulness group	22	103	5.707	321	36.44	−26.17	<0.001	5.578

M, mean; SD, standard deviation.

**Figure 1:**
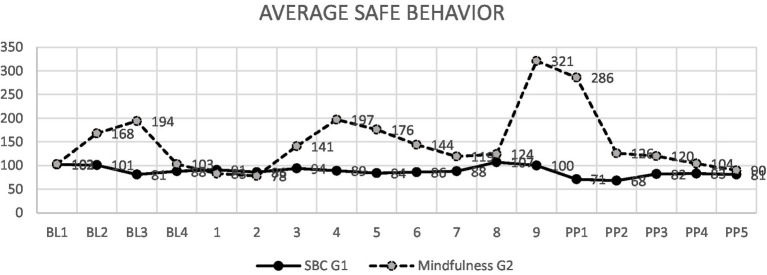
Difference between control and experimental groups per week in the three periods: baseline, application of the program, and post program.

In the evaluation between the groups, it can be observed that, during the baseline that lasted 4 weeks, no statistically significant differences were found between the two groups, but in the evaluation of the last session it can be observed that there are statistically significant differences between both groups (Table 2). This can also be seen in the graph that shows the averages of both groups recorded weekly, it can be observed that both groups start in a similar way, finding differences in both groups during the application of the program, but in the end the groups return to similar results, concluding that the program should continue to be applied to generate more stable behaviors over time (Figure 1; see Table 3).

**Table 2:** Analysis of the differences between the control (BBS) and experimental (mindfulness) groups in safe behaviors.

Subscale/group	*N*	Pre test					Post test				
		*M*	SD	*t*	*p*	*d*	*M*	SD	*t*	*p*	*d*
BBS group	22	88	42.98	1.62	0.119	0.341	100	15.684	26.126	<0.001	7.877
Mindfulness group	22	103	5.707				321	36.444			

M, mean; SD, standard deviation.

**Table 3:** Averages of safe behaviors per week of the control and experimental group in the three periods: baseline, application of the program, and post program.

		Baseline (4)				Program (9)									Post Program (5)				
		BL1	BL2	BL3	BL4	1	2	3	4	5	6	7	8	9	PP1	PP2	PP3	PP4	PP5
BBS group	G1	102	101	81	88	91	86	94	89	84	86	88	107	100	71	68	82	83	81
Mindfulness	G2	103	168	194	103	83	78	141	197	176	144	119	124	321	286	126	120	104	90

LB, baseline value per week; PP, schedule of publications per week.

## Discussion

Evidence indicates that mindfulness is very useful when it comes to reducing risky behaviors at work. As in this research, where the impact of the mindfulness program in the reduction of risky behaviors and, therefore, incidents and accidents can be evidenced. These results are confirmed thanks to research by Valley and Stallones (2017), where they found that mindfulness reduces cognitive failures in the workplace, and therefore increases staff safety behaviors, having the benefit of reducing workplace accidents.

Based on research carried out by Goilean et al. (2020), it is postulated that mindfulness would help workers to acquire a greater awareness of their true values and needs, improving job satisfaction through the mediated effect of emotion regulation.

The results obtained in the case of the mindfulness program allow us to conclude that the application of an intervention program based on mindfulness does significantly influence the reduction of accidents in the mining company, finding significant differences between the control and experimental groups with which we worked. These results would test the study’s hypothesis; in addition, the findings would have similarities with a broad base of similar studies (Liang et al., 2022; Molek and Żołnierczyk, 2018; Valley and Stallones, 2017), as well as non-experimental studies whose results refer to an association or positive influence of mindfulness with job security (Chen and Wang, 2016).

Among the studies that show similarity we can find, for example, the work of Liang et al. (2022) and Molek and Żołnierczyk (2018) who, in a company in the construction and mining sector, high-risk jobs and physical exertion respectively, concluded positive results associated with the application of a mindfulness program. However, it should be noted that Liang et al. (2022) identified that a high level of mindfulness is contradictorily associated with a rejection of the security measures implemented by the company, as well as errors and violations of security procedures. We do not know if this phenomenon happen in our research, because there was not a qualitative research.

Finally, from the results obtained we can conclude that the mindfulness program applied to the experimental group, which continued with the BBS program, gave better results than only the BBS program, applied to the control group, this can be observed from session 4 in Figure 1 where it is verified that there is a statistically significant difference between both groups. It is also important that as a limitation, it was not possible to apply only the mindfulness program without BBS, since it is not possible due to company policy. These results demonstrate that mindfulness in high-risk sectors such as mining, when used as a training tool, can help workers develop greater awareness, attention and emotional control, and these skills have a direct impact on reducing accidents and improving safety, demonstrating the proven effectiveness of mindfulness for this purpose (Vickers and Vogt, 2014). It is also necessary to mention that another limitation was the application only of the observation letter of safe behaviors and that it was not complemented with any psychological test or test and although the observation sheet reflects in a more objective way the presence of safe or unsafe behaviors, we consider that it would have been very enriching to have more sources of information. These limitations are part of the policies of the mining company, but despite them, we consider that the studies in these institutions contribute to the understanding of the working conditions that arise. Because some of these institutions are usually extreme and complex, so they are not always easily accessible and predisposed to research; this work being a contribution to this area of knowledge.

## Data Availability

The raw data supporting the conclusions of this article will be made available by the authors, without undue reservation.
